# WSG ADAPT – adjuvant dynamic marker-adjusted personalized therapy trial optimizing risk assessment and therapy response prediction in early breast cancer: study protocol for a prospective, multi-center, controlled, non-blinded, randomized, investigator initiated phase II/III trial

**DOI:** 10.1186/1745-6215-14-261

**Published:** 2013-08-19

**Authors:** Daniel Hofmann, Ulrike Nitz, Oleg Gluz, Ronald E Kates, Timo Schinkoethe, Peter Staib, Nadia Harbeck

**Affiliations:** 1 West German Study Group, Ludwig-Weber-Str. 15b, 41061 Moenchengladbach, Germany; 2 Evangelic Bethesda Hospital, Breast Center Niederrhein, Ludwig-Weber-Str. 15, 41061 Moenchengladbach, Germany; 3 Institute for Innovation and Medicine, Caramanicostr. 9A, 85551 Munich, Kirchheim, Germany; 4 St.-Antonius Hospital, Clinic for Hematology and Oncology, Dechant-Deckers-Str. 8, 52249 Eschweiler, Germany; 5 Breast Center, Department of Obstetrics and Gynecology and CCCLMU of the University of Munich, Maistr. 11, 80337 Munich, Germany

**Keywords:** ADAPT, Biomarker, Early breast cancer, Investigator initiated trial

## Abstract

**Background:**

Adjuvant treatment decision-making based on conventional clinical/pathological and prognostic single molecular markers or genomic signatures is a therapeutic area in which over-/under-treatment are still key clinical problems even though substantial and continuous improvement of outcome has been achieved over the past decades. Response to therapy is currently not considered in the decision-making procedure.

ADAPT is one of the first new generation (neo)adjuvant trials dealing with individualization of (neo)adjuvant decision-making in early breast cancer and aims to establish early predictive surrogate markers, e.g., Ki-67, for therapy response under a short induction treatment in order to maximally individualize therapy and avoid unnecessary toxicity by ineffective treatment.

**Methods/design:**

The prospective, multi-center, controlled, non-blinded, randomized, investigator initiated phase II/III ADAPT trial has an innovative “umbrella” protocol design. The “umbrella” is common for all patients, consisting of dynamic testing of early therapy response. ADAPT will recruit 4,936 patients according to their respective breast cancer subtype in four distinct sub-trials at 80 trial sites in Germany; 4,000 patients with hormone receptor positive (HR+) and HER2 negative disease will be included in the *ADAPT HR+/HER2-* sub-trial, where treatment decision is based on risk assessment and therapy response to induction therapy, and 380 patients will be included in *ADAPT HER2+/HR+*. A further 220 patients will be included in *ADAPT HER2+/HR-* and 336 patients will be recruited for *ADAPT Triple Negative*. These three sub-trials focus on identification of early surrogate markers for therapy success in the neoadjuvant setting. Patients will be allocated to the respective sub-trial according to the result of their diagnostic core biopsy, as reported by local/central pathology for HR and HER2 status.

**Discussion:**

Recent trials, such as the GeparTrio, have shown that response-guided therapy using clinical response may improve outcome. For chemotherapy or HER2-targeted treatment, pathologic complete response in a neoadjuvant setting is an excellent predictor of outcome. For endocrine therapy, response to short induction treatment – as defined by decrease in tumor cell proliferation – strongly correlates with outcome. ADAPT now aims to combine static prognostic and dynamic predictive markers, focusing not just on single therapeutic targets, but also on general markers of proliferation and cell death. Biomarker analysis will help to optimize selection of subtype-specific treatment.

**Trial registration:**

ClinicalTrials.gov: *ADAPT Umbrella*: NCT01781338; *ADAPT HR+/HER2-*: NCT01779206; *ADAPT HER2+/HR+*: NCT01745965; *ADAPT HER2+/HR-*: NCT01817452; *ADAPT TN:*NCT01815242.

## Background

The two most important questions in therapy of early breast cancer to be resolved over the next decade are: *Who can safely be spared adjuvant chemotherapy?* and *Who has the maximum benefit from chemo-, endocrine and/or anti-HER2 therapy?*

The question of who can safely be spared adjuvant chemotherapy has been extensively scientifically explored and represents the basis of decision-making with respect to adjuvant systemic therapy today [1,2]. Few well evaluated prognostic tests identify patients with a low-risk profile justifying omission of chemotherapy based on a potentially low benefit [3,4]. A number of clinical trials, such as TAILORx [5], RxPonder [3], MINDACT [6], NNBC-3 [7], and WSG planB [8], are currently addressing this question.

The second question is much more difficult to resolve. The clinically most relevant predictor for adjuvant endocrine therapy response is steroid hormone receptor protein expression [9]. Similarly, benefit from anti-HER2 therapy seems to be restricted to patients demonstrating over-expression and/or amplification of the HER2/neu oncogene, accounting for about 15% of patients with breast cancer [10,11].

The strongest clinical parameter for prediction of outcome after neoadjuvant chemotherapy is the rate of pathological complete response (pCR) [12,13]. In the adjuvant setting, the recurrence score (RS) has been demonstrated to predict outcome in hormone-sensitive disease [14]. Furthermore, HER2 over-expression correlates with anthracycline sensitivity, and luminal subtypes benefit differently from taxanes [15]. Recent data, derived mainly from primary endocrine therapy and less from primary chemotherapy, indicate that early sequential evaluation of proliferation markers such as Ki-67 strongly correlates with recurrence-free survival and overall outcome [16,17].

Within the ADAPT trial, prognostic evaluation (static biomarker) and early prediction (dynamic biomarker) are combined. The prognostic profile is evaluated at the time of diagnosis in core biopsy material and a second evaluation of proliferative and apoptotic markers as well as imaging (only in the HER2+ and triple negative setting) will be done in sequential tissue samples after a short period (3 weeks) of subtype-specific induction therapy.

Since the evidence for proliferation marker Ki-67 as an early response predictor is strongest for hormone sensitive disease, Ki-67 will be used in the *ADAPT HR+/-HER2-* sub-trial to early identify responders in the intermediate-risk group (N0-1, RS 12–25), who are then considered to be sufficiently treated by adjuvant endocrine therapy alone [14,16]. Low responders and patients initially identified as high-risk for recurrence (N2-3 or N0-1 and RS ≥26) will be randomized to a chemotherapy protocol optimizing dose-dense taxane-based chemotherapy.

The *ADAPT HR+/HER2-* sub-trial is therefore a modern biomarker-based adjuvant trial, which advances the ideas of earlier trials such as TAILORx [5], NNBC-3 [7], WSG planB [8] or MINDACT [6]. Besides better definition of prognosis, it will improve early prediction with the aim to reduce over-treatment by chemotherapy.

The ADAPT trial aims to individualize therapy by integration of early dynamic response data into clinical management. In terms of an early enrichment strategy, the purpose is to spare unnecessary toxic therapies and costs without compromising patient outcome. ADAPT thus may not only help to reduce over-treatment, but also to avoid under-treatment in early breast cancer.

## Methods/design

### Study design

ADAPT is a second generation trial addressing individualization of adjuvant decision-making in early breast cancer by utilization of optimized pre-therapeutic biomarker information and early biomarker changes as determined from a second core biopsy after three weeks of subtype-specific induction therapy. ADAPT is set up as an “umbrella” trial, i.e., all patients will complete the *ADAPT Umbrella* trial (two sequential core biopsies including biomarker determination and three weeks subtype-specific induction therapy) plus one of the breast cancer subtype-specific sub-protocols (*ADAPT HR+/HER2-*, *ADAPT HER2+/HR+*, *ADAPT HER2+/HR-* or *ADAPT Triple Negative*). Each sub-trial will utilize the subtype-specific treatment to establish individualized therapy approaches and assess early therapy response. The trial is prospective, multi-centric, controlled, non-blinded, randomized, investigator initiated phase II/III, and is run at 80 German trial sites.

### Participants

The ADAPT trial population is comprised of 4,936 women with early primary breast cancer (BC) aged between 18 and 75 years old. Any tumor size (T1-T4, except inflammatory BC) and nodal status (N) is allowed. All patients will complete the *ADAPT Umbrella* protocol irrespective of their disease subtype. Based on the sample size calculations, as adapted from the primary hypotheses to meet the primary objectives of each sub-trial, 4,000 hormone receptor positive, HER2 negative patients will be included in *ADAPT HR+/HER2-*. Another 380 patients with HER2 and HR positive tumors will participate in *ADAPT HER2+/HR+*; 220 HER2+/HR- patients will be included in *ADAPT HER2+/HR-* and 336 triple negative (HER2-/HR-) patients will be treated within the *ADAPT Triple Negative* sub-trial. Allocation to the sub-trials depends on the result for hormone receptor and HER2 status from diagnostic core biopsy as determined by local pathology. For any HER2+ or triple negative patient, the local pathology result will be verified by central pathology review since significant discordance rates between local and central pathology assessment have been described [18]. Only if central pathology confirms the local result for HR and HER2 status, the patient is eligible for the respective HER2+ or triple negative sub-trial. For HR+/HER2- patients the local pathology result is acceptable for inclusion.

### Recruitment process

Patients are recruited at participating trial sites, i.e., breast centers, highly specialized gynecologic departments, or gynecological and oncological outpatient units. West German Study Group (WSG), as the study sponsor, provides specific recruitment training to the sites as part of each onsite trial initiation visit.

Patients whose diagnostic core biopsy – as standard of care – shows a histologically confirmed unilateral primary invasive carcinoma of the breast by local pathology review will be informed about the ADAPT trial and asked to participate. Each patient has to sign three informed consent forms for inclusion, i.e., one for *ADAPT Umbrella*, one for the applicable ADAPT sub-trial as well as a third one for blood and tissue sample donation. Only if all three informed consent forms are obtained, and inclusion and exclusion criteria are not violated, the patient is ready for trial registration. Patients who are not registered prior to any trial-related procedure cannot be accepted for the trial at a later time.

### Eligibility

Female patients with histologically confirmed unilateral primary invasive carcinoma of the breast aged 18–75 years old are eligible if they are candidates for (neo)adjuvant chemotherapy by conventional prognostic criteria (age, tumor size, nodal status, grade) and have no clinical evidence for distant metastasis (M0). HR and HER2 status must be known from local pathology review and a representative tumor block must be available for central pathology review, genomic signature (where applicable), and biomarker determination. Patients must not be pregnant, i.e., a negative pregnancy test (urine or serum) within seven days prior to start of induction treatment in premenopausal patients is obligatory. Written informed consent must be obtained prior to any protocol-specific procedures and must be documented together with expected cooperation and accessibility of the patients for the treatment and follow-up according to local regulatory requirements. Patients must also be able to tolerate the treatment, as indicated by normal laboratory values and proper organ function.

Patients must not have a known hypersensitivity reaction to the compounds or incorporated substances used for treatment. Prior malignancy with a disease-free survival of less than ten years (except curatively treated basalioma of the skin or pTis of the cervix uteri) as well as sequential or non-operable BC including inflammatory BC, are not allowed. Previous or concurrent treatment with cytotoxic agents for any reason without prior consultation of the sponsor (WSG) disqualifies for trial participation. Concurrent treatment with other experimental drugs or participation in another interventional clinical trial with or without any investigational not-marketed drug within 30 days prior to trial entry is prohibited. Patients indicating risk of poor compliance or not able to consent will be excluded.

**Figure 1:**
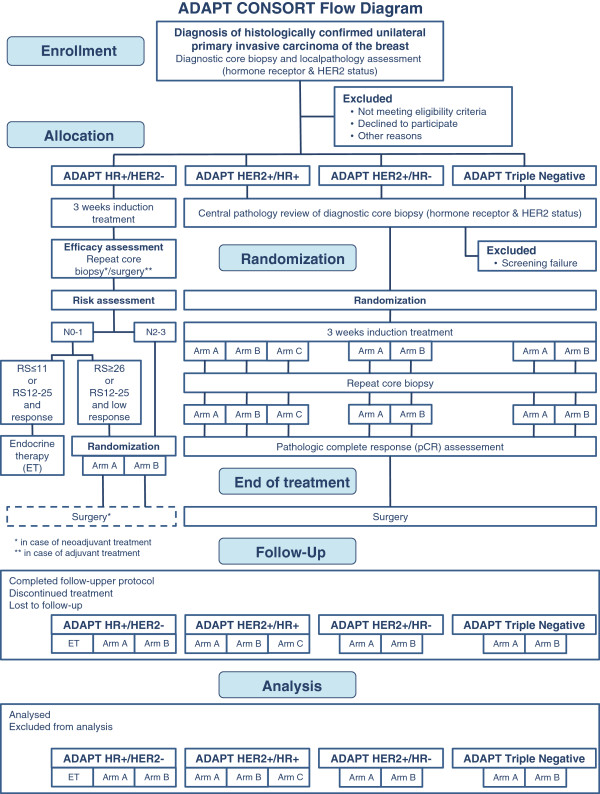
**ADAPT CONSORT flow diagram.** Patients are allocated to one of four distinct ADAPT sub-trials, depending on the hormone receptor (HR) and HER2 status of the tumor. Randomization is always subject to the respective sub-trial after allocation. Patients will be treated subtype-specific according to their individual disease. Follow-up is scheduled for five years following registration.

The procedure for enrollment, eligibility verification, treatment allocation, randomization and analysis is shown in Figure 1.

### Ethical considerations and approvals

The ADAPT trial is conducted in accordance with the Declaration of Helsinki [19], ICH-GCP and all applicable German laws and requirements. The trial received a positive vote by the leading Ethics Committee (Ethics Committee of the University of Cologne, Germany) representing the Ethics Committees of each involved institution on March 29th, 2012. The Competent Authority (Bundesinstitut für Arzneimittel und Medizinprodukte (BfArM), Germany) approved the trial on November 11th, 2011.

### Consent

Any involved investigator, who is a registered member of the principal investigator’s “investigator’s group” at the approved trial sites can contact eligible patients to inform them about the ADAPT trial and discuss the details. Eligibility criteria have to be confirmed and any required baseline measures must be obtained. Eligible individuals recruited through the above strategies are provided with three patient information sheets (*ADAPT Umbrella*, ADAPT sub-trial, blood and tissue sample donation) and three informed consent forms, respectively, all of which were approved by the leading Ethics Committee in the currently applicable version at the time of recruitment.

### Therapy response assessment

Currently, assessment of early therapy response for each sub-trial will be based on Ki-67 antigen, a nuclear protein associated with cell proliferation, which can be measured by immunohistochemistry [17]. Within the ADAPT trial, Ki-67 will be determined by central pathology for standardization of the response assessment. The measurements are performed from the diagnostic core biopsy tumor sample and the repeat core biopsy or surgical sample. Optimal therapy response is defined as a drop of Ki-67 to or below 10%.

### Treatment

#### ADAPT Umbrella

**Figure 2:**
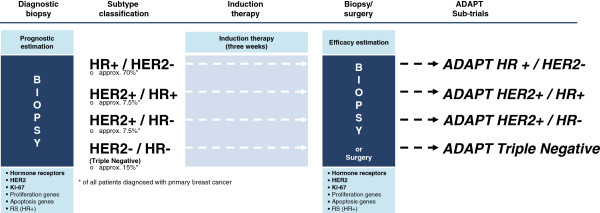
***ADAPT Umbrella *trial design.** Patients are included in the trial based on the results of the diagnostic core biopsy and local pathology for histologically confirmed tumors. Based on the subtype classification, patients are allocated to the respective sub-trial and start subtype-specific induction therapy for three weeks. Central pathology assessment includes hormone receptors, HER2 and Ki-67. For HR+ tumors, an initial RS is determined by Oncotype DX®. Following three weeks of induction therapy, efficacy estimation is done using repeat core biopsy or surgical specimen (in case of adjuvant treatment; HR+/HER2- only). The *ADAPT Umbrella* comprises two sequential tumor samples and the three week subtype-specific induction therapy. Further subtype-specific therapy is subject to the ADAPT sub-trials.

The *ADAPT Umbrella* includes the determination of Ki-67 baseline proliferation in the standard of care primary diagnostic core biopsy. Subsequently, the subtype-specific induction therapy will be applied for a short-term three-week treatment. Lastly, a repeat core biopsy or the tumor sample from surgery (in case of adjuvant treatment) will be obtained for efficacy estimation. This completes the *ADAPT Umbrella*. In the repeat core biopsy or surgery specimen, proliferation marker Ki-67 will be measured again in order to compare the pre- and post-therapeutic proliferation, which will then be interpreted either as good or low therapy response (Figure 2).

#### ADAPT HR+/HER2-

**Figure 3:**
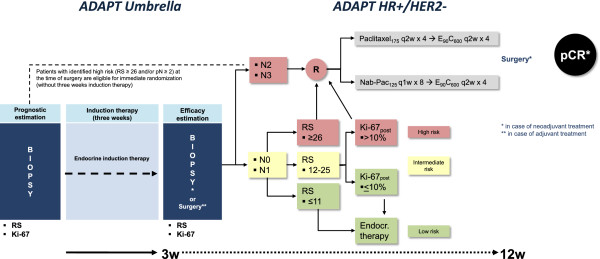
***ADAPT HR+/HER2- *trial design.** Patients with HR+/HER2- disease receive three weeks of endocrine induction therapy according to current AGO guidelines. After pathologic assessment patients are classified according to RS risk groups (low/intermediate/high risk of recurrence). Low-risk is defined as N0-1 RS ≤11 and will be allocated to adjuvant endocrine therapy alone. Definition of intermediate-risk is based on N0-1 and RS 12–25. Patients with early therapy response as measured by Ki-67_post_ ≤10% also receive endocrine therapy only. Intermediate-risk patients with low therapy response and Ki-67_post_ >10% are randomized to one of the two chemotherapy arms just like all high-risk patients, i.e., N2-3 or N0-1 and RS ≥26. Patients at high risk (pN ≥2 or RS ≥26) with clear need for chemotherapy may omit induction therapy and undergo immediate randomization.

Patients with HR+/HER2- disease receive endocrine therapy as their induction treatment. Additionally, these patients are classified according to their individual risk of recurrence. For this purpose, nodal status is used to discriminate between low (N0-1) or high (N2-3) risk patients. Any N0-1 patient will also obtain an Oncotype DX® to evaluate the genomic signature of the tumor by the 21-gene RS. Patients with RS 0–11 are classified as low-risk and are further treated just by adjuvant endocrine therapy according to AGO guidelines. Patients at high risk according to RS (≥26) are randomized to chemotherapy arm A or B. Patients with intermediate risk (RS 12–25) are treated according to their individual therapy response as determined by Ki-67 within the two sequential core biopsies. Patients with a Ki-67 drop to or below 10% after induction therapy remain under endocrine therapy only. Patients with post-therapeutic Ki-67_post_ >10% are randomized to chemotherapy arm A or B. If chemotherapy is indicated, it can either be applied within the adjuvant or neoadjuvant (for down-staging) setting at the investigator’s choice, depending on the individual tumor characteristics (Figure 3).

Subtype-specific induction therapy: endocrine therapy (according to current AGO guidelines, i.e., premenopausal: Tamoxifen (20 mg, daily); postmenopausal: aromatase inhibitors (Letrozole (2.5 mg, daily), Anastrozole (1 mg, daily) or Exemestane (25 mg, daily)) at investigator’s choice).

Adjuvant endocrine therapy: Continue as described for subtype-specific induction therapy.

Chemotherapy: Arm A: Paclitaxel 175 mg/m^2^, q2w × 4 → Epirubicin 90 mg/m^2^ + Cyclophosphamide 600 mg/m^2^, q2w × 4, versus Arm B: nab-Paclitaxel 125 mg/m^2^, q1w × 8 → Epirubicin 90 mg/m^2^ + Cyclophosphamide 600 mg/m^2^, q2w × 4.

#### ADAPT HER2+/HR+

**Figure 4:**
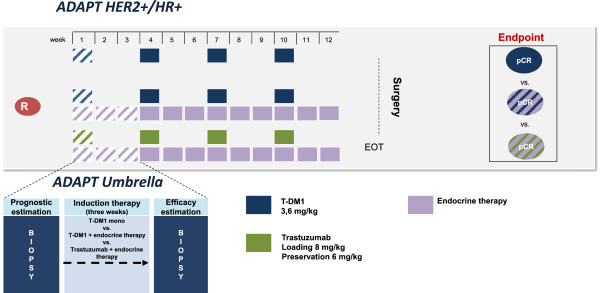
***ADAPT HER2+/HR+ *trial design.** HER2+/HR+ patients receive either T-DM1 monotherapy or T-DM1 in combination with endocrine therapy or Trastuzumab in combination with endocrine therapy as induction therapy according to their randomization. Randomization is only allowed after HR and HER2 status are confirmed by central pathology. The randomized regimen is applied in the neoadjuvant setting for twelve weeks followed by surgery.

HER2 positive patients with at least one positive HR (progesterone (PR) and/or estrogen receptor (ER)) are eligible for *ADAPT HER2+/HR+*, and will be randomly assigned to induction therapy after confirmation of receptor status by central pathology review. These patients are either treated by Trastuzumab emtansine (T-DM1) monotherapy or T-DM1 plus endocrine therapy or Trastuzumab plus endocrine therapy. Within this sub-trial, the induction therapy is continued after the repeat core biopsy (Figure 4).

Subtype-specific induction therapy and further neoadjuvant therapy:

Arm A: T-DM1 monotherapy 3.6 mg/kg body weight, q3w × 4, versus Arm B: T-DM1 3.6 mg/kg body weight, q3w × 4 + endocrine therapy (according to current AGO guidelines at investigator’s choice), versus Arm C: Trastuzumab 8 mg/kg body weight loading dose; 6 mg/kg body weight maintenance dose, q3w × 4 + endocrine therapy (according to current AGO guidelines at investigator’s choice).

#### ADAPT HER2+/HR-

**Figure 5:**
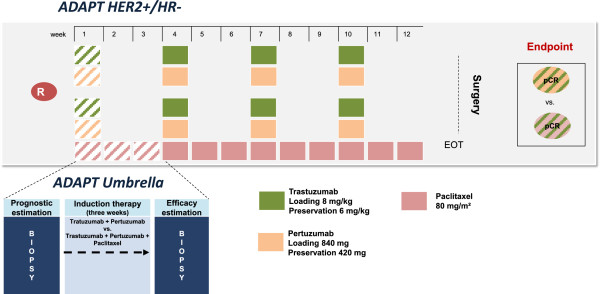
***ADAPT HER2+/HR- *trial design.** HER2+/HR- patients receive either dual anti-HER2 blockade with Trastuzumab and Pertuzumab or dual anti-HER2 blockade with Trastuzumab and Pertuzumab and chemotherapy backbone with Paclitaxel for induction therapy, which is subject to randomization. Randomization is only applicable after HR and HER2 status are confirmed by central pathology. The randomized regimen is applied in the neoadjuvant setting for twelve weeks followed by surgery.

Patients with HER2 positive tumors, but negative hormone receptors (ER and PR) are eligible for the *ADAPT HER2+/HR-* sub-trial, if receptor status is confirmed by central pathology review. Eligible patients are randomized to treatment with either Trastuzumab plus Pertuzumab or Trastuzumab plus Pertuzumab and Paclitaxel chemotherapy (Figure 5).

Subtype-specific induction therapy and further neoadjuvant therapy:

Arm A: Trastuzumab 8 mg/kg body weight loading dose; 6 mg/kg body weight maintenance dose, q3w × 4 + Pertuzumab 840 mg loading dose; 420 mg maintenance dose, q3w × 4, versus Arm B: Trastuzumab 8 mg/kg body weight loading dose; 6 mg/kg body weight maintenance dose, q3w × 4 + Pertuzumab 840 mg loading dose; 420 mg maintenance dose, q3w × 4 + Paclitaxel 80 mg/m^2^ body surface area, q1w × 12.

#### ADAPT Triple Negative

**Figure 6:**
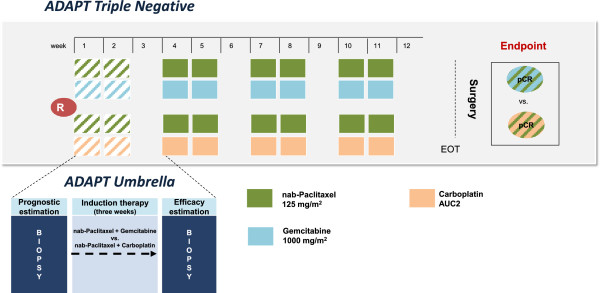
***ADAPT Triple Negative *trial design.** Triple negative (HER2-/HR-) patients are treated with either nab-Paclitaxel and Gemcitabine or nab-Paclitaxel and Carboplatin for induction therapy, which is subject to randomization. Randomization is only applicable after HR and HER2 status are confirmed by central pathology. The randomized regimen is applied in the neoadjuvant setting for twelve weeks followed by surgery.

Triple negative patients who are eligible for *ADAPT Triple Negative* after central pathology review are randomized to either nab-Paclitaxel plus Gemcitabine or nab-Paclitaxel plus Carboplatin (Figure 6).

Subtype-specific induction therapy and further neoadjuvant therapy:

Arm A: nab-Paclitaxel 125 mg/m^2^, day 1 and day 8, q3w × 4 + Gemcitabine 1,000 mg/m^2^, day 1 and day 8, q3w × 4, versus Arm B: nab-Paclitaxel 125 mg/m^2^, day 1 and day 8, q3w × 4 + Carboplatin AUC 2 (mg/mL) × min, day 1 and day 8, q3w × 4.

### Follow-up

Timing of follow-up visits is based on the date of registration. Follow-up visits are scheduled at months 9, 12, 15, 18, 24, 30, 36, 42, 48, 54 and 60 (corresponding to follow-up recommendations) after registration or until relapse to document:

– Event-free survival (EFS)

– Overall survival (OS)

– Further therapy (and/or endocrine treatment/treatment with Trastuzumab)

– Long-term toxicities

– Relapse (local relapse)

– Second primary malignancy

– First treatment for metastatic breast cancer or second primary malignancy

– Results for biopsy of distant metastases (if feasible)

– Yearly evaluation of lifestyle parameter

Patients completing follow-up month 60 are followed for survival once a year thereafter. Patients who relapse or suffer from second primary malignancy will only be followed for survival.

### Outcome assessments

#### Primary objectives ADAPT umbrella trial

The primary objective of the *ADAPT Umbrella* trial is identification of a responder sub-population within intermediate and high-risk groups in any BC subtype, which due to therapy has a comparable outcome to HR+/HER2- patients with RS ≤11 (low-risk group), i.e., 94% EFS. The low-risk group is defined as the gold standard, since this group has the best survival expectation across all BC subtypes.

#### Secondary objectives ADAPT Umbrella trial

Secondary objectives of the *Umbrella* trial include:

– Disease-free (DFS) and OS in corresponding groups

– OS

– Toxicity

– Cost-effectiveness

– Distant disease-free survival (DDFS)

– Local and regional relapse-free survival (LRFS and RRFS)

#### Primary objectives ADAPT HR+/HER2- sub-trial

The primary objectives of the *ADAPT HR+/HER2-* trial are:

– Prospective comparison of EFS in patients with intermediate risk by RS (12–25) and response to induction therapy vs. patients with low-risk (N0-1 and RS ≤11); both groups receive endocrine therapy only.

– Prospective comparison of 5-year EFS of nab-Paclitaxel 125 mg/m^2^ q1w × 8 versus Paclitaxel 175 mg/m^2^ q2w × 4, both followed by conventionally dosed E_90_C_600_ × 4 q2w chemotherapy regimens; patients with intermediate-risk (N0-1, RS 12–25 and low response to induction therapy) or high-risk (N2-3 or N1-2 and RS ≥26) will be treated with chemotherapy.

#### Primary objectives ADAPT HER2+/HR+ sub-trial

The primary objectives of *ADAPT HER2+/HR+* are:

– Comparison of the pCR rates in patients with HER2+/HR+ breast cancer (HER2+/HR+: HER2+/ER+ and/or PR+) treated by pre-surgical T-DM1 with or without standard endocrine therapy or Trastuzumab with endocrine therapy given for a total of twelve weeks.

– Evaluation of dynamic testing (based on proliferation/apoptosis changes in repeated biopsy and imaging by MRI) after three weeks of treatment as a surrogate parameter for response (pCR (residual cancer burden (RCB) 0–1) or resistance/low response (RCB II-III or progressive disease).

#### Primary objectives ADAPT HER2+/HR- sub-trial

The primary objective of *ADAPT HER2+/HR-* sub-trial is:

– Definition of a biomarker (profile) characterizing “responders” to dual anti-HER2 blockade of Trastuzumab and Pertuzumab that have similar pCR rates as patients treated with identical dual anti-HER2 blockade + taxane backbone.

#### Primary objectives ADAPT Triple Negative sub-trial

The primary objectives of ADAPT Triple Negative comprise:

– Comparison: pCR in nab-Paclitaxel + Carboplatin vs. nab-Paclitaxel + Gemcitabine.

– Comparison: pCR in responders vs. non-responders.

### Central pathology/Oncotype DX®

Logistics are a key component of the ADAPT trial. Any tumor sample, either from the diagnostic core biopsy or the repeat second core biopsy or surgery will be shipped for review to the central pathology laboratory (director: Prof. Dr. Kreipe), situated at the Institute of Pathology at the Medical University of Hannover in Germany.

The greatest challenge are the logistics for tumor samples in *ADAPT HR+/HER2-*, because prior to central pathology assessment of the diagnostic core biopsy material the tumor sample will be shipped to the United States for Oncotype DX® testing in order to receive the individual patient’s RS. RS is routinely determined within ten business days, including shipment. After RS determination, the tumor sample will be sent back to Germany for central pathology assessment, which is usually performed within five business days and reported back to the trial sites via online electronic case report form (e-CRF).

### Sample size and power calculation

#### ADAPT umbrella trial

For *ADAPT Umbrella*, prospective comparisons will be conducted between two groups (involving all four sub-trials) defined as follows:

*ADAPT Umbrella* Experimental Group (AU-EG) comprising:

– AU-EG1: Intermediate-risk (N0-1 and RS 12–25) patients with early response in HR+/HER2- disease (receiving no chemotherapy);

– AU-EG2: Patients with pCR in HER2+/HR+ disease;

– AU-EG3: Patients with pCR in HER2+/HR- disease;

– AU-EG4: Patients with pCR in triple negative disease.

*ADAPT* Reference Group (A-RG) comprising:

– Low-risk HR+/HER2- (N0-1 and RS 0–11) patients.

According to trial recruiting targets and using evidence-based estimates of RS fractions, 28% of HR+/HER2- patients (n = 1,120) will be classified in the AU-EG1 group (intermediate-risk with early response and no chemotherapy); 170 patients are expected in the remaining AU-EG groups (pCR). The reference group A-RG (low-risk, no chemotherapy) will contain an expected 640 patients, i.e., 16% of expected 4,000 HR+/HER2- patients randomized to the trial.

As an exploratory analysis (no formal hypothesis; the purpose of the trial is to explore this assumption more thoroughly in order to develop some specific hypothesis or prediction that can be tested in future research), non-inferiority of the experimental group (AU-EG) compared to the reference group (A-RG) will be tested with regard to EFS. Assuming 94% 5-year EFS in both groups, and defining “non-inferiority” in terms of a δ = 3.2% margin, (i.e., no worse than 90.8% 5-year EFS in the experimental group), a one-sided test with α = 5% will have 80% power (taking expected dropouts into account).

#### ADAPT HR+/HER2- sub-trial

For patients not receiving chemotherapy in this sub-trial, the ADAPT reference group A-RG is the same as in the *Umbrella* trial: low-risk HR+/HER2- (N0-1 and RS 0–11) patients. As stated above, this reference group has expected size n = 640 and expected 5-year EFS of 94%. The primary hypothesis to be tested in this sub-trial is non-inferiority of the AU-EG1 experimental group (N0-1/RS 12–25 responders) compared to the reference, with regard to the primary endpoint EFS. This experimental group has an expected size of n = 1,120 (about 28% of HR+/HER2- patients). With a non-inferiority margin of δ = 3.3%, (i.e., no worse than 90.7% 5-year EFS in AU-EG1), a one-sided test with α = 5% will have 80% power (taking expected dropouts into account).

High-risk, HR+ breast cancer patients are randomized to chemotherapy with either nab-Paclitaxel → Epirubicin + Cyclophosphamide (EC) (experimental group) or dose dense Paclitaxel → EC (active control group). In these patients, the aim is to demonstrate at a 95% confidence level that the EFS of patients in the experimental group is non-inferior to EFS of patients in the active control. This design takes into account the clinical relevance based on the expected safety profile and other advantages of nab-Paclitaxel → EC compared to the active control for important patient subgroups.

The power of the trial is computed using a 5-year EFS estimate of 84% in the active control group, supposing that the true hazard rates are equivalent, and allowing a non-inferiority margin of δ = 3.85 % in 5-year EFS (hazard ratio 1.269). With 2,240 patients expected in this part of the trial and 5% dropout (i.e., 2,128 patients actually evaluated), the trial power is 80% to reject the null hypothesis (inferiority) by a one-sided test with α = 5%.

#### ADAPT HER2+/HR+ sub-trial

Patients are randomized to either of two T-DM1 arms (with or without endocrine therapy) or to a Trastuzumab arm with endocrine therapy. The study aims to test the hypothesis of higher pCR separately in each of the T-DM1 arms. Assuming a dropout rate of 5%, a test collective of approximately 300 HER2+/HR+ patients is defined (assumed 75% based on evidence from the GeparQuattro, NOAH and I-SPY 1 trials and others).

A 10% rate of pCR in the Trastuzumab + endocrine therapy arm is expected. In each T-DM1 containing arm, 25% pCR is assumed. Assuming α = 5% (one-sided), an improvement of this magnitude is detectable with at least 80% power in each of the T-DM1 containing arms compared to the Trastuzumab + endocrine therapy arm.Interim analysis on correlation between changes in the sequential biopsy and pCR rates in the first 130 patients (run-in phase) is planned during recruitment.

#### ADAPT HER2+/HR- sub-trial

In this two-arm, randomized neoadjuvant trial in HER2 positive, HR negative patients, the issue is whether an early-response biomarker can be found that defines a subgroup of patients (“responders”) with an “enhanced” rate of pCR, that is, their rate is sufficiently favorable such that chemotherapy could be omitted in a clinical context. To this end, two arms are considered: Patients randomized to Arm A (“no-chemotherapy arm”) receive Trastuzumab + Pertuzumab; patients randomized to Arm B (“chemotherapy arm”) receive Trastuzumab + Pertuzumab + taxane backbone. For the primary hypothesis, pCR of early responders of Arm A is compared with pCR of Arm B as a whole.

Omission of chemotherapy is formalized by a test of the hypothesis of non-inferiority (with respect to the primary endpoint, pCR) between “responders” of the “no-chemotherapy arm” (A) and all patients of the “chemotherapy arm” (B). The power analysis is based on this hypothesis test.

If the primary hypothesis is confirmed (i.e., the null hypothesis for the primary endpoint is rejected), a secondary analysis will be performed in which pCR will be estimated in three subgroups defined via the combination of treatment and biomarker status. In these three subgroups, the pCR rates with confidence intervals adjusted for multiple testing by the Sidak correction (α = 0.017) to achieve a family-wise error rate no larger than α = 0.05. Sample size computations for the primary hypothesis were performed under the following assumptions:

– Overall pCR in arm A (no chemotherapy): 30%

– pCR in arm B (chemotherapy): 60%

– Fraction of arm A with biomarker positive, i.e., “responders”: 40%

– pCR in responder subset of arm A: 60%

– α = 0.05, 80% power, non-inferiority δ = 23% (regarding pCR)

Design ratio n_A/n_B = 2.5

– n_A = total subjects in arm A: 143

– n_B = total subjects in arm B: 57

– Total subjects in both arms: 200 + 10% dropouts

The ratio n_A/n_B = 2.5 is intended to keep as many patients as possible in arm A to test possible secondary endpoints within that group while also satisfying the requirements on overall power for the primary endpoint.

#### ADAPT Triple Negative sub-trial

In the *ADAPT Triple Negative* sub-trial, comparison of pCR is to be carried out in responders vs. non-responders and in nab-Paclitaxel + Carboplatin vs. nab-Paclitaxel + Gemcitabine. The comparisons involve testing of two co-primary hypotheses. The family-wise error α is controlled for multiple testing at the level 0.05; an unequal allocation of α is used for the two tests.

Regarding comparison of responders and non-responders: the overall proportion of pCR with these medications in TNBC is estimated at about 25%. This proportion includes both responders (about 60% of TN patients) and non-responders (about 40% of TN patients). A difference of 17% between pCR proportion of responders and pCR proportion of non-responders would be of clinical interest. A one-sided test with α = 0.01 is performed for a difference of 17% between responders and non-responders (e.g., 31.8% versus 14.8%). Under the above assumptions, this test would reject the null hypothesis (no difference in proportions) with better than 80% power with a total of n = 320 patients (336 including drop-outs), taking into account variability in the true percentage of responders. Regarding the medication comparison: there is indirect evidence that pCR could be higher in the B (nab-Paclitaxel + Carboplatin) arm. A two-sided test with α = 0.04 will be performed for a difference of 15% in pCR proportion between arm B (nab-Paclitaxel + Carboplatin) and arm A (nab-Paclitaxel + Gemcitabine). A difference of this magnitude would be clinically relevant for triple negative BC. Under these assumptions, the null hypothesis can be rejected with at least 80% power with n = 320 patients (336 including dropouts).

### Randomization

Randomization to any treatment is always subject of the respective sub-trial. In any case, patients are only randomized after confirmation of eligibility, consenting and registration. Any patient, who was not randomized prior to first treatment administration (if applicable) will not be accepted for the trial at a later time. Randomization is performed centrally according to a stratified permuted block design. The randomization form has to be filled in online (e-CRF). After completion, it must be printed, signed by an investigator and faxed to the study coordinator. The study coordinator selects the applicable randomization sheet according to stratification parameters and randomizes the patient via the e-CRF. Stratification will be performed by study center and nodal status (p or cN0, N1, N2, N3). Further stratification parameters for *ADAPT HR+/HER2-* include treatment (neoadjuvant/adjuvant) and RS group (high-risk vs. intermediate and low response).

### Data management

The data are captured via a password-protected online e-CRF, which is based on Microsoft SQL Server and corresponding Microsoft SQL databases for secure repository of trial data. For data security reasons different roles with distinct competences in e-CRF use are available. There are three distinct parties which have access to the e-CRF: first the trial sites for data capture, second the central pathology for result reporting and data capture, and third clinical research associates for data monitoring and quality assurance. Trial sites can have access as “documentalists” with read and write rights or “investigator” with read-only rights to overview the onsite data capture. Investigators have to verify the captured data on a regular basis by signature. The central pathology has access only to the central pathology e-CRF page to report the result of central pathology review. This e-CRF report is considered as source data and can only be handled by the central pathology. Monitors have access to any e-CRF page and can comment on any erroneous e-CRF page and return them for correction to the trial site’s documentalist. Any change or correction to e-CRF pages is tracked and marked by the system. Once the monitor has verified the correctness of e-CRF pages, these pages are irrevocably closed for further data capture. From then, any new data can only be entered via written data clarification forms to the trial data manager.

### Data analysis

All sub-trials are designed with a run-in phase and a main phase. The run-in phase will comprise a small percentage of patients per sub-trial in order to evaluate the assumptions made across the sub-trials, e.g., Ki-67 cut-offs, risk group distribution or response rates. For *ADAPT HR+/HER2-,* the first 400 out of 4,000 patients will be included in the run-in phase. For the other sub-trials, the following distributions for the run-in phase are planned: *ADAPT HER2+/HR+* 130 of 380; *ADAPT HER2+/HR-* 75 of 220 and *ADAPT TN* 130 of 336.

An intention-to-treat (ITT) analysis will be conducted for all randomized comparisons. Analyses that are not randomized comparisons will be conducted among the eligible patients (per-protocol (PP) population).

### Population analysis

Populations to be analyzed are:

– ITT and PP populations (ITT1 and PP1) in low and intermediate-risk HR+/HER2- disease treated with no chemotherapy.

– ITT and PP populations (ITT2 and PP2) in high-risk HR+/HER2- (high-risk and N0-1/intermediate with no response to endocrine therapy) disease treated with chemotherapy.

– ITT and PP populations in HER2+/HR+ (ITT3 and PP3), HER2+/HR- (ITT4 and PP4) and triple negative (ITT5 and PP5) disease.

– Safety population within low-risk HR+/HER2- (SP1), high-risk HR+/HER2- (SP2), HER2+/HR+ (SP3), HER2+/HR- (SP4) and TNBC (SP5), who have started their allocated treatment (at least one dose of the drug).

Analysis of EFS and OS will be performed in the ITT population, defined as the population of all randomized patients analyzed in the treatment group they were assigned to. Analysis of EFS and OS will also be performed in the eligible patient populations, defined as the ITT population patients without patients who were randomized, but were not eligible for the trial according to the inclusion and exclusion criteria.

Final analysis (pCR) in ITT3–5 and PP3–5 will be performed after all patients within HER2+ and triple negative subgroups have completed surgery. Exploratory analysis in patients of ITT2 and PP2, who underwent neoadjuvant chemotherapy, will be performed also after completion of surgery. Prognostic factor analysis will be defined at the time of end analysis. Both local and central data will be used. The safety analysis will be conducted on all patients who started at least one infusion of the study treatment.

Within the *ADAPT HR+/HER2-* sub-trial, the assumed subgroup distributions will be evaluated. The current estimates are low-risk (20%); intermediate-risk with response to endocrine therapy (35%); intermediate-risk with low response to endocrine therapy (15%); and high-risk (30%). Taking into account an estimated 20% N2-3 as high-risk, effectively 16% of patients are expected to be low-risk; 28% intermediate-risk with response; 12% intermediate-risk with low response; and 44% high-risk. Thus, 44% of registered patients (all with an indication for chemotherapy based on clinical assessment) will be allocated to the “ADAPT-guided low-risk” subgroup and 56% of patients to the “ADAPT-guided high-risk” subgroup.

### Independent data safety monitoring committee (IDMSC)

Overall safety will be assessed on an ongoing basis during the conduct of the trial. The IDMSC will monitor cumulative safety data at least once every six months during the course of the trial. In addition, data on serious adverse events and deaths will be monitored by the IDMSC at least once every three months.

## Discussion

Currently, adjuvant therapy indications in early HR+/HER2- breast cancer are mainly based on the individual prognostic profile as determined by clinical-pathological factors (tumor size, nodal status, grade, age), prognostic genomic signatures (e.g., RS) or protein markers such as uPA/PAI-1 [20,21]. These factors allow for identification of a patient subgroup at such a low risk of recurrence that absolute reduction of recurrence risk by adjuvant chemotherapy would only be marginal [20]. However, even if modern molecular risk stratification tools are used, there remains a large group of patients at intermediate risk for whom the magnitude of benefit from adjuvant chemotherapy is unclear [22].

The primary goal of ADAPT is early response assessment, based on Ki-67, in all subtypes of breast cancer as clinically defined by hormone receptor status and HER2 over-expression. ADAPT is in line with recent trials, such as POETIC [23], WSG planB [24], I-SPY 1 [9,25] and GeparTrio [26], aiming at incorporation of new diagnostic methods for both patient and subtype-specific treatment of breast cancer. Each of these trials focused on early molecular marker assessment, while ADAPT moves the field forward by covering all subtypes of early breast cancer.

The POETIC trial addresses hormone sensitive disease only. The trial assesses the influence of neoadjuvant endocrine therapy (randomization to two weeks of pre-surgical endocrine therapy vs. none) on proliferation in postmenopausal estrogen receptor positive women in a prospective setting [23]. Yet, in contrast to ADAPT, no further treatment decision is based on the early treatment response.

The I-SPY 1 trial focuses on the predictive value of early serial imaging and serial biomarker profiles for pathologic complete response and recurrence-free survival in a population with tumors ≥3 cm receiving neoadjuvant anthracycline-based chemotherapy followed by taxanes at the discretion of the investigating trial site [25]. Central pathology assessment was limited to HER2 status, whereas HR status was determined locally [9]. In the multivariate analysis, the molecular signatures providing additional information after consideration of clinical pathological factors and pCR were Mammaprint, wound healing signature, p53 mutation signature and PAM50. No therapeutic consequences were drawn from the results, while both the treated population and the treatment were heterogeneous [9,25]. In contrast, ADAPT uses central assessment of HR, HER2, and Ki-67 since high levels of discordance have been reported for local vs. central pathology assessment [18]. ADAPT will thus provide high quality assurance standards. Although logistics for the central pathology assessment are challenging, the central pathology results are reported back to the trial sites within five business days on average.

Within the phase III GeparTrio trial early clinical therapy response to neoadjuvant chemotherapy is taken into account for further therapy decision-making in all breast cancer patients being candidates for chemotherapy [27]. The trial involved 2,090 patients for comparison of Docetaxel + Doxorubicin + Cyclophosphamide (TAC) × 6 versus TAC × 8. Early response in this trial was assessed by ultrasound after two courses of TAC. A complete response was defined as no sonographic signs of disease. Response was regarded as partial if the product of the two largest perpendicular diameters of the primary tumor was reduced by 50% or more. If the reduction in tumor size was less than 50% or tumor growth did not exceed 25%, the response was documented as no change. More growth or the occurrence of a new lesion were classified as progressive disease. Patients with no change at the first control (after 2 × TAC) were randomized to further TAC versus navelbine/capecitabine. Patients receiving non-cross-resistant chemotherapy in the case of no change had a significant survival benefit from changing the regimen [26]. In retrospective subgroup analyses, the effect was mainly limited to patients with luminal A and luminal B tumors [27]. Although, ADAPT does not include change of the regimen in case of low response, except for endocrine therapy in the HR+/HER2- setting, it will potentially identify patients were change to a non-cross-resistant regimen could be beneficial to reduce mistreatment and improve individual outcome.

WSG planB is a prospective randomized phase III trial comparing anthracycline-free chemotherapy (Docetaxel + Cyclophosphamide (TC) × 6) versus an anthracycline-based standard (EC → Doc) [24]. Oncotype DX® was used in HR+/HER2- patients to reduce over-treatment in the low-risk group (RS ≤11 (18%)), which was recommended not to take chemotherapy [24]. For HR+/HER2- disease ADAPT includes the same patient population as planB and recommends endocrine therapy only in low-risk patients as defined by RS ≤11. In the intermediate-risk population (RS 12–25), treatment-decision is made on the basis of early response to endocrine therapy measured by baseline and sequential Ki-67 assessment. Patients defined as early responders are spared chemotherapy and receive endocrine therapy only. A pooled analysis of both trials will allow important comparisons of conventional prognostic parameters with a new generation of prognostic tools.

In summary, ADAPT surpasses any of the recent biomarker-driven trials in terms of complexity and potentially also of clinical significance given its inclusion of all breast cancer subtypes. Together with RxPonder [3], TailorX [5] and WSG planB [8], ADAPT will prospectively validate the clinical utility of the RS by Oncotype DX®. To bridge the gap between prognosis and prediction of therapy success there is a need to regard individual response to therapy, which has so far not been included systematically into the clinical decision-making procedure. Potentially, the additional evaluation of early therapy response will elucidate the unclear magnitude of benefit from chemotherapy in intermediate-risk patients, who are a substantial proportion of patients in early breast cancer [24]. The definition of therapy response is primarily based on two consecutive Ki-67 evaluations. Nevertheless, the ADAPT design allows identification of further molecular markers and profiles with a potentially even stronger correlation to therapy response and thus may lead to replacement of the currently used markers.

Early prediction of outcome based on therapy response may be a powerful instrument to identify over- or under-treatment and therefore may become a unique tool to identify potential mistreatment. It could spare unnecessary toxicity and costs if these modern enrichment strategies were integrated to management of early breast cancer.

In hormone-sensitive disease, data are already available identifying the early drop of proliferation marker Ki-67 under endocrine treatment as a potent early surrogate for EFS [14,16]. Presumably, these data are mature enough to spare chemotherapy to those patients with hormone-sensitive disease, who are at intermediate risk, but highly responsive to endocrine therapy. The hypothesis is that good responders are sufficiently treated by adjuvant endocrine therapy only, whereas patients with low response need additional treatment such as (neo)adjuvant chemotherapy.

In HER2 over-expressing and triple negative disease, the data on early response prediction are less mature [28]. Therefore, the evaluation of the dynamic test is the main objective defined for these sub-protocols. Without interfering with this primary endpoint, innovative new compounds such as T-DM1, Pertuzumab (HER2+) or nab-Paclitaxel (HR+/HER2- and triple negative) have been implemented in the sub-trials. Together with the dynamic test the ADAPT concept will provide important and rapid early feedback for future planning of large-scale phase III trials. Especially in the context of a vast number of new biological agents and the high costs associated with registration and marketing approval there is a major challenge for both new generation trial designs by academia and drug development by the pharmaceutical industry.

The *ADAPT Umbrella* trial brings together the results of dynamic testing acquired in the different subgroups. It will allow identification of patients within all breast cancer subtypes, who have intermediate to poor prognosis according to conventional criteria, but excellent outcome due to high therapy efficacy. Due to the applied therapy response assessment, this population is identified as early as possible within the ADAPT trial. Consequently, the main hypothesis is to spare further unnecessary therapy for these patients, without compromising the individual outcome. The low responding population (i.e., potentially mistreated) may become another major future focus of ADAPT. Further adaptive strategies may be warranted to generate valuable information about this subgroup and improve their outcome.

The umbrella protocol design is a new powerful tool, especially in heterogeneous diseases like breast cancer, in order to apply one common therapy concept to all disease subtypes. Virtually any patient diagnosed with primary breast cancer can be included in the trial and may benefit from the early response assessment and further individualization of therapy by taking into account not only the conventional tumor characteristics, but also the individual response to the applied therapy. Last, but not least, the ADAPT concept is flexible enough to incorporate new sub-trials or to implement highly innovative treatments as they arise from early clinical or translational research.

In conclusion, trial sites and patients can equally benefit from the ADAPT concept by establishing one common trial concept for all breast cancer subtypes. The additional central pathology assessment assures and improves the diagnostic quality for the trial sites, the individual patient as well as the clinical scientists. Finally, access to new and promising substances for specific and targeted treatment of the different breast cancer subtypes as well as flexible trial design, make WSG ADAPT an innovative, while clinically highly relevant trial. It will contribute to the goal of therapy individualization for each individual patient by maximizing treatment efficacy in early breast cancer at the same time. The importance of this investigator initiated trial for clinical management is recognized by the fact that for the first time, payers in the German healthcare system are working together with an academic study group as well as partners from the diagnostic and pharmaceutical industry, in order to support the WSG ADAPT trial. Especially, since ADAPT is an investigator initiated trial where the monetary possibilities are limited, the advantages are outstanding.

## Trial status

The ADAPT trial is currently recruiting patients. The first patient in the *ADAPT HR+/HER2-* trial was registered on May 10th, 2012. By August 2013, 510 patients have been recruited for *ADAPT HR+/HER2-,* 55 patients for the *ADAPT HER2+/HR+* sub-trial and 8 for the *ADAPT Triple Negative* sub-trial. *ADAPT HER2+/HR-* is not yet recruiting. 44 of 80 sites are actively recruiting and a further 36 sites were recently approved by the leading Ethics Committee and will be initiated within the next months.

## Abbreviations

ADAPT: Adjuvant dynamic marker-adjusted personalized therapy; A-RG: ADAPT Reference Group; AU-EG: ADAPT Umbrella Experimental Group; BC: Breast cancer; eBC: Early breast cancer; e-CRF: Electronic case report form; EFS: Event-free survival; ER: Estrogen receptor; GCP: Good clinical practice; HER2: Human epidermal growth factor receptor 2; HR: Hormone receptor; IDMSC: Independent data monitoring and safety committee; ICH: International conference on harmonization; ITT: Intention-to-treat; LN: Lymph nodes; nab-Paclitaxel: Nanoparticle albumin-bound-paclitaxel; OS: Overall survival; Pac: Paclitaxel; PAI-1: Plasminogen activator inhibitor-1; pCR: Pathological complete response; PP: Per protocol; PR: Progesterone receptor; RCB: Residual cancer burden; RS: Recurrence score; T-DM1: Trastuzumab Emtansine; TAC: Docetaxel + Doxorubicin + Cyclophosphamide; uPA: Urokinase-type plasminogen activator.

## Competing interests

UN received honoraria for lectures from Genomic Health, Inc. and for consulting from AOK Rheinland/Hamburg. NH received honoraria for consulting and lectures from Roche and Celgene. She also received honoraria for lectures from Genomic Health, Inc. and Amgen. REK is an immediate family member of NH. PS received honoraria for consulting and lectures from Roche, Celgene and Amgen. DH, OG and TS declare they have no competing interests.

## Authors’ contributions

DH provided the draft of the manuscript and developed the administrative, regulatory and operational parts of the trial protocol. UN, NH and OG developed the scientific as well as medical parts of the trial protocol and contributed to, reviewed and approved the manuscript. REK provided the statistics for the trial protocol and the manuscript and reviewed and contributed to it. TS and PS contributed to and reviewed the manuscript and provided scientific feedback. All authors read and approved the final manuscript.
